# Supplementary material for: Optimizing CO2 field flooding during sternotomy: In vitro confirmation of the Karolinska studies
Source: PLoS One. 2024 Jan 9;19(1):e0292669. doi: 10.1371/journal.pone.0292669 (PMC10775975; doi:10.1371/journal.pone.0292669)
Supplement: S1 Table — β: coefficients of selected parameters, SE: standard error, df: degree of freedom, t: t value, p: p-value, SD(ID): standard deviation of random effects variances. (DOCX) [file pone.0292669.s003.docx]

| **Fixed effects:** | | | | | |
| --- | --- | --- | --- | --- | --- |
|  | β | SE(β) | df | t | p |
| **Left ventricle** | | | | | |
| (Intercept) | 77.46 | 2.30 | 73 | 33.7 | **<.0001** |
| Position 6 | -4.75 | 2.32 | 73 | -2.0 | **<.05** |
| Position 12 | 3.72 | 2.32 | 73 | 1.6 | 0.1 |
| Flow rate 2 L/min | -3.76 | 2.01 | 73 | -1.9 | 0.1 |
| Andocor | 2.59 | 2.47 | 73 | 1.0 | 0.3 |
| Drainage catheter | -15.80 | 3.34 | 73 | -4.7 | **<.0001** |
| Temed | -7.69 | 2.47 | 73 | -3.1 | **<.01** |
| Bent | 18.83 | 2.01 | 73 | 9.4 | **<.0001** |
| **Underneath heart** | | | | | |
| (Intercept) | 77.95 | 2.23 | 74 | 35.0 | **<.0001** |
| Position 6 | -4.98 | 2.41 | 74 | -2.1 | **<.05** |
| Position 12 | 3.97 | 2.41 | 74 | 1.6 | 0.1 |
| Flow rate 2 L/min | -3.88 | 2.09 | 74 | -1.9 | 0.1 |
| Drainage catheter | -18.12 | 3.36 | 74 | -5.4 | **<.0001** |
| Temed | -7.92 | 2.41 | 74 | -3.3 | **<.01** |
| Bent | 19.37 | 2.09 | 74 | 9.3 | **<.0001** |
| **Random effects:** | | | | | |
| SD(ID) | | | | | |
| **Underneath heart** | | | **Left ventricle** | | |
| 8.6017 | | | 7.7981 | | |
