# Supplementary material for: Optimizing CO2 field flooding during sternotomy: In vitro confirmation of the Karolinska studies
Source: PLoS One. 2024 Jan 9;19(1):e0292669. doi: 10.1371/journal.pone.0292669 (PMC10775975; doi:10.1371/journal.pone.0292669)
Supplement: Attachment — Submitted filename: Response to Reviewers.docx [file pone.0292669.s004.docx]

Dear Editor in-Chief,

We would like to thank you for accepting to reconsider a revised version of our manuscript entitled: “Optimizing CO_2_ field flooding during sternotomy: in vitro confirmation of the Karolinska studies”. We really appreciate the efforts of the academic editor and the reviewers in improving our manuscript and the positive feedback provided during the review process. We modified the script the best we could following the reviewers’ comments and suggestions, and we offer the following point-by-point replies:

Reviewer #1

The reviewer’s comments captured very well our intention to deliver insights, while knowing that we cannot provide perfect answers for the clinical situation. We are grateful for his wise assessment that our study should only be interpreted and judged within this intended scope.

Reviewer #2

We are grateful for the reviewer’s statement that our study may help alleviate inadequate knowledge about the effects of various factors on CO_2_, which could lead to more consistent or equipoised clinical studies in the future.

1. *The introduction is too long and should be shortened and simplified for this relatively limited dataset and report.*

We removed some sentences from the introduction while keeping the key message.

2. *To increase the interest, I would suggest including data regarding the effect of the use of pump suckers on the CO2 levels as this manuscript has fairly limited data but serves as a basis for additional testing (as you indicated) but some additional variables would make this basic report much more interesting*

Investigation of the effect of pump suckers is interesting as indicated by the reviewer and essential according to us. We already did it in a follow-up study, which will be presented in an upcoming article. We decided to split into two articles because the type of analysis performed is completely different and cannot be merged into one statistical analysis. For the study here under review, we assessed steady conditions to create insights in what can ideally be achieved and the whole complicated multi-factorial statistical analysis was performed on the mean CO_2_ values obtained during the plateau phase (equilibrium). While for the effect of pump suckers, we investigated some dynamic parameters to capture the transient effects such as rising time to reach the maximum CO_2_ plateau, drop in CO_2_ concentration due to suction, and drop and recovery percentages and times when suction is turned off and then on again. We hope that the upcoming manuscript will satisfy the curiosity of the reviewer as it complements this first article.

3. *There is going to be a large number of variables ultimately affecting the CO2 levels measured in this report, such as pump suckers, the surgeon and assistants and others. For instance, what was the ventilation system of the room used? OR requirements include air exchange rates which generally exceed those of normal rooms and the presence of OR lights overhead, etc. can affect the local air flow; did you perform these tests in a functional OR room or somewhere else and how would you propose to evaluate the room ventilation effect and light positions on CO2 levels?*

We previously tested the effect of ventilation both in an actual OR (with also the surgeon wearing sensors) by moving the OR table relative to the downflow and in our lab by adjusting the ceiling fans. Preliminary observations with CO_2_ sensors and Schlieren visualization showed that the impact is negligible, so we performed the final study in a laboratory with disabled ventilation. We did not test the effect of heating caused by lamps, but we assume that is also negligible compared to the factors that were under investigation. Effect of surgeon and assistant was also tested in a previously published study and in our OR simulation, and we excluded it from this one due to the focus on steady conditions.

4. *Do you have a plan to monitor CO2 levels during actual surgery? How would you propose to do that? Is it practical and if so, would low CO2 levels inappropriately pressure the surgeon to change techniques?*

As the reviewer guessed, we do indeed plan to monitor CO_2_ during actual surgery, and we are developing a prototype device to do so. Reporting on this device is premature, as it is still work in progress and therefore we considered this issue as beyond the scope of the here reported study.

5. *What about using animal models, such as porcine models to better understand how having real tissue in an accurate anatomic configuration affects CO2 levels?*

We considered the option of using animal models as a next step now that we have reached insights and we can better filter the experimental settings that would yield interesting results. Since the here presented extensive set of conditions took several days of model measurements, this would be impossible to achieve in a single animal, thus weakening the statistical power. An animal model would also eliminate the option of obtaining the Schlieren insights. This would be a completely different study and beyond our initial scope, hence we choose to not discuss this in our manuscript.

6. *Overall the English grammar is acceptable but there are clear opportunities to improve this aspect and should be carefully reviewed by someone fluent in English.*

We thank the reviewer for this suggestion, and we had the manuscript reviewed for language.

Reviewer #3

1. *For me, one major questions remains unclear, is there an upper level of CO2 that is dangerous? Is it describe? If yes, what are the sides effects? This reflection should be a part of the discussion.*

As suggested by reviewer #3 we added a paragraph about side effects of CO_2_ insufflation in the Discussion section. The maximum CO_2_ concentration that can be reached in the atmosphere in and around the chest is 100%. The potential danger comes from its absorption into the blood, which can and should be easily compensated by an experienced perfusionist by increasing the fresh gas flow through the oxygenator. Monitoring of blood gases around the start of CO_2_ field flooding is part of our routine clinical practice and we have had no issues with excessive pCO_2_ thanks to this simple compensation method.

We also noted that reviewer #3 responded with “I don’t know” to the assessment of the appropriateness of our statistical analysis. We would like to point out that this seemingly simple laboratory parameter study has baffled highly experienced clinical statisticians due to the combination of factors, the repetitions, and the fact that our “population” consists of one patient model which reduces variability but is contrary to how clinical trials are set up. We eventually teamed up with 3 professional experts and had several discussions on approaches and models. We are convinced that the eventual presentation reflects the best way to analyze these data, but there is not unique “correct” way for this, just different acceptable approaches.

The funding statement was corrected as requested and the cover letter was amended accordingly. Copyrighted images in Table 1 were replaced with our own. While Figures 1, 4 and 5 were kept the same because they contain only original pictures taken by our group. However, tables containing images are now labeled as figures and the numbering of figures and tables was adapted.

Additionally, we made the necessary changes to comply with the PLOS ONE style templates.

We would like also to request a change in the authors’ list. We would like to add a new co-author, Alberto Pagnamenta, who was previously mentioned in the Acknowledgments. After careful consideration, we conclude that Alberto Pagnamenta satisfies the requirements to be a co-author of this manuscript. All co-authors agree with this change.

We respectfully hope that it might be possible for you to take the revised version of our manuscript into consideration. We appreciate the comments and suggestions of the reviewers and thank them for helping us to improve our manuscript.

Kindest regards,

Stefanos Demertzis, MD

Department of Cardiac Surgery, Istituto Cardiocentro Ticino, Lugano, Switzerland

Faculty of Biomedical Sciences, Università della Svizzera Italiana, Lugano, Switzerland

Email: stefanos.demertzis@eoc.ch
